# 926. You Got Burned, Again: A Study on Burn Recidivism at an Academic Burn Center

**DOI:** 10.1093/jbcr/irag033.466

**Published:** 2026-04-06

**Authors:** Daniel R Streetman, Alexa DeRegnaucourt, Victoria Lee, Adam Hutchinson, Andrew Villegas, Uzair A Qazi

**Affiliations:** University of Cincinnati Medical Center, Cincinnati, OH; University of Cincinnati College of Medicine, Cincinnati, OH; University of Cincinnati College of Medicine, Cincinnati, OH; University of Cincinnati College of Medicine, Cincinnati, OH; University of Cincinnati College of Medicine, Cincinnati, OH; University of Cincinnati College of Medicine, Cincinnati, OH

## Abstract

**Introduction:**

Trauma recidivism refers to the incidence of new and unrelated injuries after an initial trauma. Pre-existing comorbidities such as mental illnesses and substance abuse have been linked to higher rates of trauma recidivism. These findings are further supported by subsequent studies where intervention programs utilizing cognitive behavior therapy and peer mentoring have been successful in decreasing recidivism rates. Yet, the phenomenon of burn recidivism is seldom described in the literature, marking a key area of interest where interventions may benefit high-risk populations. Here, we examine the characteristics and outcomes of burn recidivists (RC) compared to non-recidivists (NRC) at a single institution’s burn center.

**Methods:**

Patients evaluated in the emergency department and discharged or admitted to the burn unit from 2010 to 2025 were evaluated using a single academic institution’s burn registry. 2496 patients were identified, with 1435 related to new burns. Patients with an isolated inhalation injury, Stevens-Johnson Syndrome, or Toxic Epidermal Necrolysis were excluded. Eight patients were identified as RC by the occurrence of a second burn. Wilcoxon rank-sum or Fisher’s exact test was performed to compare demographics, pre-existing comorbidities, toxicology screens upon admission, burn TBSA%, and morbidity across RC and NRC groups.

**Results:**

No significant differences were found between age, gender, or drug usage across both cohorts (*p*<.382, *p*<.452, *p*<.08544411583). Upon burn analysis, the NRC group was found to have higher TBSA and partial thickness involvement compared to the RC group (*p*<.0063, *p*<.0352). RC were found to have a higher number of comorbidities, particularly with limitations to activities of daily living (ADL) (*p*<.02, *p*<.040). Within the RC group, half of the second burn encounters originated from an identical mechanism as the first. Additionally, the mechanism for eight of the sixteen burn encounters in the RC group was due to smoking while on supplemental oxygen.

**Conclusions:**

Low rates of burn recidivism and the multifactorial nature of this phenomenon may complicate the ability to identify specific risk factors for burn recidivists. Our exploratory findings suggest that an increased number of coexisting health conditions, particularly concerning ADLs, may be associated with risk of recidivism. Burns resulting from smoking on supplemental oxygen may be a key area of interest where interventions may benefit recidivism rates in the future. However, further research using data from multiple institutions is warranted to strengthen and identify the characteristics of high-risk populations for burn recidivism.

**Applicability of Research to Practice:**

This study demonstrates that burn recidivism has an overall low prevalence, and patients that are actively smoking or have limitations on ADLs may benefit from further interventions to decrease recidivism rates.

**Funding for the study:**

N/A.

